# The parent-adolescent dyad in autism spectrum disorder: which level of burden?

**DOI:** 10.1192/j.eurpsy.2025.1149

**Published:** 2025-08-26

**Authors:** R. Jbir, F. Cherif, R. Masmoudi, M. Chaabene, J. Boudabous, I. Feki, F. Guermazi, J. Masmoudi

**Affiliations:** 1 psychiatry ‘A’ department; 2 Child psychiatry department, Hedi Chaker university hospital, Sfax, Tunisia

## Abstract

**Introduction:**

Autism Spectrum Disorder (ASD) is a neurodevelopmental disorder that begins in early childhood. For the young people with ASD Adolescence is a particular time of life, in which they are facing physical and mental changes and for their parents who have to cope with the challenges associated with their child’s development. The effect of these different stressors on caregivers represents the burden.

**Objectives:**

The aim of our study was to assess the level of burden experienced by parents of adolescents with ASD and to investigate the factors associated with it.

**Methods:**

Our study was cross-sectional, descriptive and analytical conducted in the form of a survey during the month of May 2024. It concerned the parents of adolescents with ASD followed at the “Erraihan” therapeutic farm in Sfax. All data relating to adolescents and parents were processed solely by the interviewer.

The form included a section for collecting data relating to the adolescent and the parent, and one to measure caregiver burden via the Zarit Burden Interview (ZBI).

**Results:**

Our population was comprised of 43 parents with a sex-ratio equal to The mean age of parents was 50.6 **±**7.93 years, with extremes of 36 and 81 years.

As for the adolescents, the mean age was 17.79 ±2.29 years (min=13; max=20).

Psychometric assessment of parental burden showed that the mean Zarit score was 24.6±20.14, with extremes of 0 and 48. The results showed that 62.80% of parents felt a burden of severe intensity.

The level of parental burden increased with the presence of at least one history of somatic illness (p=0.03).

Similarly, severe burden was significantly associated with the presence of more than two children in the family (p=0.02) and the presence of sibling rivalry (p=0.04).

With regard to adolescent characteristics, the level of severe burden was significantly associated with male gender (p=0.03) and rank as the eldest sibling (p=0.01).

The presence of severe burden in the parents was significantly associated with the presence of self-aggressive behavior in the adolescent (p=0.02).

With regard to therapeutic characteristics, the use of risperidone and sodium valproate was significantly associated with an absent to moderate level of burden(p= 0.01 for both cases).

Similarly, the adolescent’s previous integration into school was significantly associated with an absent to moderate level of burden (p=0.01).

**Conclusions:**

The results suggest that parents of adolescents with autism need more support to cope with the burden generated by the adolescent’s behavior. It is therefore necessary to adjust the support provided to parents in order to optimize the quality of care provided for their adolescents.

**Disclosure of Interest:**

None Declared

